# Supplementary material for: The Systems Biology Research Tool: evolvable open-source software
Source: BMC Syst Biol. 2008 Jun 29;2:55. doi: 10.1186/1752-0509-2-55 (PMC2446383; doi:10.1186/1752-0509-2-55)
Supplement: Additional file 1 — SBRT Archive. An archive of the current version of the Systems Biology Research Tool. [file 1752-0509-2-55-S1.zip › sbrt-1.4.0/doc/developers_guide/api/sbrt/shell/mng/KernelProcessManager.html]

KernelProcessManager


|  |  |  |
| --- | --- | --- |
| **Overview** \| **Package** \| **Class** \| **Use** \| **Tree** \| **Deprecated** \| **Index** \| **Help** | |  |
| **PREV CLASS**   **NEXT CLASS** | **FRAMES**    **NO FRAMES**     **All Classes** |  |
| SUMMARY: NESTED \| FIELD \| CONSTR \| METHOD | DETAIL: FIELD \| CONSTR \| METHOD |  |


---


## sbrt.shell.mng Interface KernelProcessManager<K,V>

**Type Parameters:**: `K` - the key type.: `V` - the value type.

**All Superinterfaces:**: ProcessManager<K,V>

**All Known Implementing Classes:**: CatalystDeletionManager, CdHarFluxVectorManager, CdHarManager, CorrelationManager, CvCatalystDeletionManager, CvFbaOptManager, CvMsFluxIntervalManager, CvObjectiveFunctionManager, CvRevRxnFluxIntervalManager, CvRxnDeletionManager, CvSimpleFluxIntervalManager, CycleIdManager, ExtremeCurrentManager, FbaManager, FbaOptManager, FbaSolverManager, FluxActivityManager, FluxCapManager, FluxPlasticityManager, InitialPointManager, IntervalComparisonManager, IntervalVariationManager, KendallTauManager, LinearSolverManager, MannWhitneyUManager, MsFluxIntervalManager, MsFluxomeReducerManager, NetworkInfoManager, ObjectiveFunctionManager, PathIdManager, RandomConstraintsManager, RandomEnvManager, RandomFluxVectorManager, RandomObjectiveManager, ReverseRxnFluxIntervalManager, RxnDeletionManager, SimpleFluxIntervalManager, SingleElementUnionManager, SlpCycleIdManager, StoichVariationManager, StrictSingleElementUnionManager, SvCdHarFluxVectorManager, SvInitialPointManager, VariableParticipationManager, VectorComparisonManager, WwFluxomeReducerManager

---

``` public interface KernelProcessManager<K,V> extends ProcessManager<K,V> ```

This interface is used to represent process managers.
A process manager is used to control a `KernelProcess`.
The manager is responsible for combining inputs from
different sources and passing this information to a
`KernelProcess` in a way it can understand.

A process manager is created from a `ManagerInput`
object. Process managers require a minimum set of key-value
pairs to construct their associated `Process`.
In addition to this minimum set, a set of optional
key-value pairs may also be allowed. If the provided `ManagerInput`
contains any additional keys beyond the set of required and optional keys,
an `ApplicationException` will be thrown.

**Author:**
:   This interface was written and documented by
    Jeremiah Wright while in the Wagner lab.

---

| **Method Summary** | |
| --- | --- |
| `KernelProcess<?>` | `getProcess()`             Returns the process this process manager controls. |

**Methods inherited from interface sbrt.shell.mng.ProcessManager**

`check, execute, execute, getOptionalKeys, getRequiredKeys, setInput, setInput`

**Method Detail**

### getProcess

```
KernelProcess<?> getProcess()
```

:   Returns the process this process manager controls.
    This method helps maintain the integrity of the shell and
    kernel by contractually forcing a process to exist. In other words,
    this method prevents a process manager from performing
    process-level operations.

    :   **Returns:**: the process this process manager controls.


---


|  |  |  |
| --- | --- | --- |
| **Overview** \| **Package** \| **Class** \| **Use** \| **Tree** \| **Deprecated** \| **Index** \| **Help** | |  |
| **PREV CLASS**   **NEXT CLASS** | **FRAMES**    **NO FRAMES**     **All Classes** |  |
| SUMMARY: NESTED \| FIELD \| CONSTR \| METHOD | DETAIL: FIELD \| CONSTR \| METHOD |  |


---
